# In-depth proteomic analysis of boar spermatozoa through shotgun and gel-based methods

**DOI:** 10.1186/s12864-018-4442-2

**Published:** 2018-01-18

**Authors:** Jean M. Feugang, Shengfa F. Liao, Scott T. Willard, Peter L. Ryan

**Affiliations:** 1 Department of Animal and Dairy Sciences, Mississippi State University, Mississippi State, MS 39762 USA; 2 Department of Biochemistry, Molecular Biology, Entomology and Plant Pathology, Mississippi State University, Mississippi State, MS 39762 USA; 3 Department of Pathobiology and Population Medicine, Mississippi State University, Mississippi State, MS 39762 USA

**Keywords:** Fertility, Fertilization, Semen, Spermadhesins, Swine

## Abstract

**Background:**

Mature spermatozoa contain numerous epididymal and seminal plasma proteins, which full identification through high-throughput technologies may allow for a better understanding of the sperm biology. Therefore, we conducted a global proteomic analysis of boar spermatozoa through shotgun and gel-based methodologies.

**Results:**

The total proteins were extracted from mature spermatozoa and subjecsted to proteome analyses. Functional analyses of gene ontology representations and pathway enrichments were conducted on the shotgun dataset, followed by immunology and gene expression validations. Shotgun and gel-based approaches allowed the detection of 2728 proteins and 2123 spots, respectively. Approximately 38% and 59% of total proteins were respectively fully and partially annotated, and 3% were unknown. Gene ontology analysis indicated high proportions of proteins associated with intracellular and cytoplasm localizations, protein and nucleic acid binding, hydrolase and transferase activities, and cellular, metabolic, and regulation of biological processes. Proteins associated with phosphorylation processes and mitochondrial membranes, nucleic acid binding, and phosphate and phosphorous metabolics represented 77% of the dataset. Pathways associated with oxidative phosphorylation, citrate cycle, and extra-cellular matrix-receptor interaction were significantly enriched. Protein complex, intracellular organelle, cytoskeletal parts, fertilization and reproduction, and gap junction pathway were significantly enriched within the top 116 highly abundant proteins. Nine randomly selected protein candidates were confirmed with gel-based identification, immunofluorescence detection, and mRNA expression.

**Conclusions:**

This study offers an in-depth proteomic mapping of mature boar spermatozoa that will enable comparative and discovery research for the improvement of male fertility.

**Electronic supplementary material:**

The online version of this article (10.1186/s12864-018-4442-2) contains supplementary material, which is available to authorized users.

## Background

Spermatozoa are haploid cells produced in the testis with a specific shape allowing them to carry and deliver paternal materials to the oocyte. Their biology has a multifaceted array of genetic, proteomic and metabolic differences, which unpredictable interactions with one another could influence the sperm function and consequently, male fertility. Numerous studies have focused on both liquid and solid phases of semen ejaculates to investigate the multifactorial causes of male subfertility or infertility [1–4]. With regard to spermatozoa, various methods are being used to shed light on every aspect of their structure and functionality. Motility and motion kinematics of spermatozoa are routinely assessed with computerized systems for objective, highly accurate and repeatable evaluations [5–7], while viability parameters such as the integrity of the plasma, acrosome, and mitochondrial membranes are evaluated with vital fluorescent dyes [8, 9]. Still, these critical assessments do not always translate into a consistent prediction of semen quality and fertility.

Unpredictable interactions of spermatozoa with secretions found in the male reproductive tract make their biology more complex. Indeed, spermatozoa are released within the seminiferous tubules upon their production and maintain continuous interactions with surrounding secretions of the genital tract (testes and epididymis) throughout their longitudinal migration within the epididymis lumen and accessory sex glands (prostate, cowper’s gland and seminal vesicles). These protein-based interactions contribute to shaping the sperm structure and influence their fertility [10–14]. The quantity and quality of these proteins may vary significantly with the physiological and health status of the male, and only a global investigation of spermatozoa’s total proteins and their interactions can help understand and address problems associated with sperm manipulation and fertility [15].

This past decade has been marked by the rapid development of high-throughput technologies allowing fast discoveries of the wealth of sperm molecules (i.e., RNA transcripts and proteins). The use of high-throughput techniques for either profiling or comparative studies of spermatozoa has been successful in various species such as the bull [4, 16–19]**,** mice, rat [20, 21], men [15, 22], and the worm *Caenorhabditis elegans* [23]. Various proteomic platforms exist and a wide range of protein numbers have been detected in spermatozoa across and within species. To date, the best estimate figures are found to be between 2500 and 3000 proteins [15, 24–26] using either shotgun or gel-based analytical methodologies.

Each methodology has advantages and disadvantages [24–27]. The shotgun methodology is a bottom-up approach requiring an initial digestion of the crude protein extract into peptides to allow the identification of a greater number of proteins, but information concerning their likely post-translational modifications are lost. In contrast, the gel-based approach can afford a means for discovery, but several critical variables such as the protein extraction methods, the relative amount of proteins within extracts, the magnitude of the pH gradient in the first dimension, the molecular weight range in the second dimension, the means by which proteins will be stained, and repeatability remain and limit the amount of detectable proteins in samples [24, 28, 29].

So far, the rationale of selecting one technical approach over another has been driven by the objective of the study [30], and both methodologies have been used independently or in combination in many species for either profiling or discovery studies [25, 26]. Contrary to many other species [22, 31], gel-based proteomics have been the preferred methods in boar spermatozoa studies [32–34]. In spite of the usefulness of the gel-based approach, only limited numbers of proteins have been identified and the full profiling of boar spermatozoa is necessary for an in-depth comprehension of their biology to speed biomarker discoveries in an effort to improve fertility outcomes in swine. A similar approach has been recently undertaken to boost the proteome of boar seminal plasma from less than 100 [10, 35], to several hundred (536) identified proteins [36].

Here, we employed both shotgun (nanoLC-MS/MS) and gel-based (2-DE) proteomic approaches, followed by functional bioinformatics (functional annotations, enrichment, and biological pathways) analyses to provide a panoramic overview of the proteome of mature boar spermatozoa. Validations were conducted with in situ immunofluorescence, westernblotting, and mRNA gene expression. This study confirms the power of the shotgun proteomic at generating large amounts of proteins compared to the gel-based method, while providing the first and most comprehensive proteome of mature boar spermatozoa. Additionally, the bioinformatic analyses indicate the likely importance of oxidative phosphorylation, citrate (TCA) cycle, and ECM-receptor interaction and gap junction pathways on sperm function. Generated dataset will be useful to better understand the biology of boar spermatozoa that is needed to further improve fertility diagnosis and prognosis of males and develop appropriate strategies for efficient post-collection handling of semen, particularly in swine.

## Methods

### Semen collection and sperm purification

Twenty-four semen ejaculates (3 per boar) of eight fertile Landrace boars (Table 1) of approximately 1.5 years old were purchased (International Boar Semen; Eldora, IA). Each semen ejaculate was extended with the Beltsville Thawing Solution (BTS: Minitube of America – MOFA; Verona, WI) at the boar stud facilities and overnight-shipped to our laboratory, in three different occasions (one ejaculate/shipping). Twenty-four extended semen doses of sperm motility averaging 85 ± 2.7% (mean ± sem) at collection, were used in this study. Sperm motility were evaluated upon reception in our laboratory (75 ± 3.8%) and spermatozoa were purified through a discontinuous percoll gradient as previously reported [37]. After several washes with cold phosphate buffered-saline (PBS), sperm samples devoid of contamination (e.g. extender components and somatic cells such as leukocytes and testicular cells) were stored in aliquots of 100 × 10^6^ spermatozoa per ejaculate and per boar at − 80 °C. Samples were arranged for proteomics (three different ejaculate aliquots per boar; Additional file 1: Figure S1), immunodetection (immunofluorescence and westernblotting), and gene expression (RT-PCR) analyses.

**Table 1:** Boar reproductive characteristics

Expected Progeny Difference (EPD) class	Values (mean ± sem)
Number of offspring Born Alive (NBA)	12.9 ± 1.1
Total Litter weight (LWT; pound)	184.2 ± 4.2
Days at 250 pounds (D/250)	151.5 ± 0.5
Sow Productivity Index (SPI)	110.5 ± 4.4

### Shotgun proteomic

For each boar (*n* = 8), spermatozoa of three different ejaculates were pooled (3 × 100 × 10^6^ spermatozoa) and eight independent pools were used for analyses at the Institute for Genomics, Biocomputing and Biotechnology, Mississippi State University.

#### Sample cleanup method

Total protein of each boar (3 × 100 × 10^6^ = 300 × 10^6^ spermatozoa/boar) was extracted using the complete radioimmunoprecipitation assay (RIPA; Thermo Fisher Scientific Inc., Waltham, MA) buffer [38] and quantified (Bradford assay kit). Isolated protein samples were digested with trypsin and resulting peptide soup were desalted using a peptide macrotrap (TR1/25108/52; Michrom BioResources, Auburn, CA). After removal of the digestion buffer (2% and 90% acetonitrile, 0.1% formic acid), samples were cleaned using a strong cation exchange (SCX) trap (Michrom TR1/25108/53) to remove any detergents or other polymers that can interfere with MS/MS analysis. After further cleaning procedures through the SCX trap, the dried matrix of salt crystals and peptides were resuspended in 20 μl of 5% acetonitrile, 0.1% formic acid and transferred to a low retention autosampler vial for deconvolution via reverse phase, high-pressure liquid chromatography.

#### Nanospray LC/MS method

Each sample was loaded on a BioBasic C18 reversed phase column (Thermo 72,105-100,266) and flushed for 20 min with 5% acetonitrile (ACN), 0.1% formic acid to remove salts. Peptide separation was achieved using a Thermo Surveyor MS pump with a 655-min nano-HPLC method consisting of a gradient from 5% ACN to 50% ACN in 620 min, followed by a 20 min wash with 95% ACN and equilibration with 5% ACN for 15 min (all solvents contained 0.1% formic acid as a proton source). Ionization of peptides was achieved via nanospray ionization using a Thermo Finnigan nanospray source type I operated at 1.85 kV with 8 μm internal diameter silica tips (New Objective FS360-75-8-N-20-C12). High voltage was applied using a t-connector with a gold electrode in contact with the HPLC solvent. A Thermo LCQ DECA XP Plus ion trap mass spectrometer was used to collect data over the 655 min duration of each HPLC run. Precursor mass scans were performed using repetitive MS scans, each immediately followed by three MS/MS scans of the three most intense MS peaks. Dynamic exclusion was enabled with duration of 2 min and repeat counts. Once a mass is measured twice, it is added to a list to be excluded from further analysis for a predetermined amount of time, which was 2 min. This allowed the MS to collect data on different masses, while in the meantime, the mass will have eluted from the column. Dynamic exclusion allows for a more efficient and deeper sample coverage [39].

### Gel-based proteomic

For each boar (*n* = 8), spermatozoa of 3 different ejaculates were pooled (300 × 10^6^ spermatozoa) and 8 independent pools were submitted to Applied Biomics (Hayward, CA, USA) for analyses.

#### Protein lysate preparation

Total protein was extracted as described above (300 × 10^6^ spermatozoa/boar) and resuspended with 200 μl 2D lysis buffer [2 M thiourea, 7 M urea, 4% (*w*/*v*) CHAPS, 30 mM Tris-HCl (pH 8.8)]. Samples were sonicated, centrifuged at high speed, and supernatants were collected, and protein quantified and normalized to 5 mg/ml with the 2-D cell lysis buffer.

#### Isoelectric focusing (IEF) and SDS-PAGE

Protein preparations (5 mg/ml) were mixed with 2× concentrated 2-D Sample buffer [8 M urea, 4% CHAPS, 20 mg/ml dithiothreitol or DTT, 2% pharmalytes and trace amount of bromophenol blue - 0.002% (*w*/*v*) -], followed by an addition of 100 μl Destreak solution and Rehydration buffer (7 M urea, 2 M thiourea, 4% CHAPS, 20 mg/ml DTT, 1% pharmalytes and trace amount of bromophenol blue). Protein samples (350 μl = 300 μg) were placed into a strip holder for protein loading in 18 cm IPG strips. The IEF was run as recommended by Amersham BioSciences, under dark at 20 °C. Thereafter, IPG strips (pH 3-10) were incubated in a fresh made equilibration buffer 1 (50 mM Tris-HCl, pH 8.8, containing 6 M urea, 30% glycerol, 2% SDS, trace amount of bromophenol blue and 10 mg/ml DTT) for 15 min with slow shaking. The strips were transferred to a freshly made equilibration buffer 2 (50 mM Tris-HCl, pH 8.8, containing 6 M urea, 30% glycerol, 2% SDS, trace amount of bromophenol blue and 45 mg/ml Iodoacetamide) and incubated 10 min with slow shaking. Afterwards, the IPG strips were rinsed once in the SDS-gel running buffer and placed onto 12% SDS-Gel electrophorese gels and sealed with 0.5% (*w*/*v*) agarose solution (in SDS-gel running buffer). The SDS-gels were run at 15 °C and stopped until the front dye runs out of the gels.

All gels (*n* = 8) were fixed and stained with the Bio-Safe colloidal Coomassie Blue G-250 solution (Bio-Rad Laboratories, Hercules, CA).

#### Image scan and data analysis

Image scans were carried out immediately following the SDS-PAGE using Typhoon TRIO (GE Healthcare) following the manufacture’s protocol. Scanned images were analyzed by Image QuantTL software (GE-Healthcare) and subjected to in-gel analysis and cross-gel analysis using DeCyder software version 6.5 (GE-Healthcare). Spots with either protein score or total Ion (C.I. %) greater than 95 were considered significant and protein candidates were randomly selected for spot picking.

#### Spot picking, in-gel trypsin digestion, and MS

The spots of interest were picked up by Ettan Spot Picker (GE Healthcare) based on the in-gel analysis and spot picking design by DeCyder software. Gel spots were washed few times and digested in-gel with modified porcine trypsin protease (Trypsin Gold; Promega, Madison, WI). Digested tryptic peptides were desalted by Zip-tip C18 (Millipore) and eluted from the Zip-tip with 0.5 μl of matrix solution (α-cyano-4-hydroxycinnamic acid, 5 mg/ml in 50% acetonitrile, 0.1% trifluoroacetic acid, 25 mM ammonium bicarbonate) and spotted on the MALDI plate. Mass spectrometer (MALDI-TOF) and TOF/TOF (tandem MS/MS) were performed (5800 mass spectrometer; AB Sciex), and MALDI-TOF mass spectra were acquired in reflectron positive ion mode, averaging 2000 laser shots per spectrum. TOF/TOF tandem MS fragmentation spectra were acquired for each sample, averaging 2000 laser shots per fragmentation spectrum on each of the 10 most abundant ions present in each sample (excluding trypsin autolytic peptides and other known background ions).

### Protein identification and bioinformatics

Database searches were performed using the SEQUEST algorithm in Bioworks 3.3 [40] and GPS Explorer 3.5 equipped with search engine (Matrix science) to search the *Sus scrofa* databases (NCBI-nr and ENSEMBL) for resulting peptide masses and matching peptide spectra. Proteins identified with shotgun were functionally annotated (Gene ontology or GO, Enrichment, and KEGG pathway) using online tools of Agbase (www.agbase.msstate.edu; [41]) and DAVID (Database for Annotation, Visualization and Integrated Discovery; DAVID Bioinformatics Resources 6.7; https://david.ncifcrf.gov/; [42]).

### Immunodetection of selected proteins

#### Immunofluorescence for flow cytometry and microscopy

Immediately after sperm purification, aliquots of purified spermatozoa were fixed with 4% paraformaldehyde and submitted for immunofluorescence detection using standard protocol, as previously reported [43]. Briefly, suspended and fixed spermatozoa were successively permeabilized in PBS, containing 1% triton-X100 (30 min), blocked in PBS containing 0.1% Teween-20 and 0.5% BSA (30 min), incubated overnight at 4 °C with commercial primary antibodies (Santa Cruz Biotechnology, Inc., Santa Clara, CA) diluted 100× with PBS, and finally incubated 60 min with FITC-conjugated secondary antibody (1/200 dilution). Between steps, samples were washed twice by centrifugation (800×g – 5 min) and immunolabeled spermatozoa were submitted for flow cytometry evaluation (excitation of 488 nm). Sperm cells incubated without any antibodies or only the secondary antibody served as negative controls to set up the flow cytometer (FACSCalibur; Becton Dickinson; Franklin Lakes, NJ). Sperm cells were excited with a 488-nm laser source and the FITC and the Propidium iodide (PI) dyes were detected with the FL-1 (530/30 nm) and FL-2 (585/42 nm), respectively. A total of 10,000 events was evaluated for data acquisition. In parallel, aliquots of immunolabeled spermatozoa were smeared and mounted onto histology slides with DAPI containing medium. Slides were visualized under a Laser Scanning Confocal Microscope 710 (Zeiss) with a plan-apochromat 63×/1.40 Oil DIC M27 objective and images were analyzed with the ZEN 2012 SP1 (black edition) software.

#### Westernblotting

Total protein was extracted from three different ejaculates per boar (300 × 10^6^ spermatozoa) using the complete RIPA lysis buffer. After protein quantification, equal amounts of extracted proteins per each boar were pooled according to the ejaculate (*n* = 3 pools) and total of 30 μg/pool was loaded onto wells of SDS-PAGE (4-12.5% NuPAGE) gels for resolution at room temperature. In-gel proteins were transferred onto PVDF membranes and the immunoblotting procedure was performed according to the anti-rabbit WesternBreeze™ Chromogenic Detection kits (Thermo Fisher Scientific Inc.). Membranes were incubated 60 min with selected primary antibodies (1/500 dilution).

In both immunological techniques, primary antibodies raised against human aquaporin 1 (AQP-1) and 7 (AQP-7), sodium/glucose co-transporter 5 (GLUT-5), plasminogen (PLG), protamine-1 (PRM-1), and thioredoxin reductase 1 (TRXR-1) purchased at Santa Cruz Biotechnology, Inc. were used. Otherwise indicated, all procedures took place at room temperature.

### Reverse transcription-polymerase chain reaction (RT-PCR)

Total RNA were extracted from boar sperm samples using the RNeasy Mini kit (Qiagen, Carlsbad, CA) as previously described [37], with an in-column DNase digestion. Isolated RNA was reverse-transcribed into the complementary DNA (cDNA; Quantitech RT kit, Qiagen) and 0.25 μg of cDNA was used for polymerase chain reaction (PCR) with primers specifically designed to amplify water aquaporin (AQP-1, − 5, and − 11), aquaglyceroporin (AQP-3, − 7, − 9, and − 10) sodium/glucose co-transporter (GLUT-3 and GLUT-5) and spermadhesins (SPMI, AWN, AQN-1, PSP-I, and PSP-II) transcripts (Table 2). Samples were amplified in a total of 25-μl reaction volume using SYBR Green kit (Qiagen) within the Rotor-gene Q Thermal Cycler (Qiagen). The PCR conditions consisted of a hot start (95 °C-5 min), 40 cycles of amplification (95 °C-15 s and 60 °C-30 s), and a final extension (72 °C-5 min). Water was used as a template for negative control, while GAPDH was used as the housekeeping gene. All PCR products were subjected to electrophoresis on a 1.5% agarose gel and product images were documented.

**Table 2:** Primer characteristics

Gene names	NCBI Accession #	Primer sequences (5’➔3′)	Product size(bp)
Water aquaporins			
AQP-1	NM_214454	FP: CCGGCAACTCCCTTGGCCTG RP: GGGTTAATGCCGCAGCCGGT	220
AQP-5	NM_001110424	FP: TGGGCTGGCACCTGGCAATG RP: CGACTGCGGGGCCGAAAGAG	266
AQP-11	NM_001112682	FP: TGCGTGGGTGCCTTGTGGAG RP: GTGCAGCAAAGCGCTGTGGAA	150
Aquaglyceroporins			
AQP-3	NM_001110172	FP: TCGTGTGCGTGCTGGCCATT RP: ACCGGCGATGGAACCCAGGA	257
AQP-7	NM_001113438	FP: AGTGACCGGTCCCACAGCCA RP: CTGTGTGCCCCAGCCAGCAA	295
AQP-9	NM_001112684	FP: GGGCTGCAACCCTCTTTGGCA RP: TGGGCTGTAGGCCTCTGGGGA	234
AQP-10	NM_001128454	FP: GGGTCTGTGGCCCAGGCAGTA RP: TGGCCAGGGAGAAGGCTGGA	148
Spermadhesins			
AQN-1	NM_001025210	FP: CAGGCTGCTGAGATGAAGCTGGGC RP: CGCAGGCGAGGTTGAGATACGG	235
AWN	NM_213829	FP: GTCCTCAGAGACCCTCCTGGGAA RP: GGAAGGGAGAGGCACGCTGG	277
PSP-I	NM_213837	FP: GCGGTTCTTCAGGCATGACGGT RP: GTGGCCAGGAGATGGCGCTC	203
PSP-II	NM_213836	FP: ACTGCCATCCCCTGGGCCTT RP: GCCGTAGACAAAAAGACAATCGCTG	305
SPMI	NM_001031776	FP: TCCGAGACCAACGGGCAGGAC RP: TGGCTCCTTGTGGGATGCCGT	165
Glucose transporters			
GLUT-3	FJ_209733	FP: GGGGGCCTTTGGCACTCTCA RP: AAGCCCAAGAGCAGGGGCCA	117
GLUT-5	EU_012359	FP: TCGGCTCCCTCATGGTCGGC RP: TGGGCGACTTTGCTGCATCCC	118

### Statistical analyses

Sample collection was designed to minimize inter-ejaculate and inter-individual variations, with spermatozoa harvested from three independent semen collections of eight fertile boars during spring (April and May). Proteomics were run on mixed aliquots of three ejaculates, per boar and for each boar. Only the shotgun proteome datasets providing higher number of proteins were used for statistical analyses according to Pendarvis et al. [44]. Peptide search results were filtered using a decoy based statistical method in which a probability of being a false positive match was assigned to each peptide. Proteins containing at least three peptides with a *P* value (Benjamini-Hochberg correction) of 0.05 or less were retained as indicative of the confidence in protein identification and relative expression [45]. The enrichment of GO terms and KEGG pathways was identified through the Fischer’s Exact Test with Multiple Testing Correction of FDR (Benjamini-Hochberg) used to calculate the probability that the association of proteins with given biological function or pathway was not due to random chance. Cutoffs were set for *P* ≤ 0.05 and FDR ≤ 10%.

## Results

### Total protein identification using shotgun

**Fig. 1:**
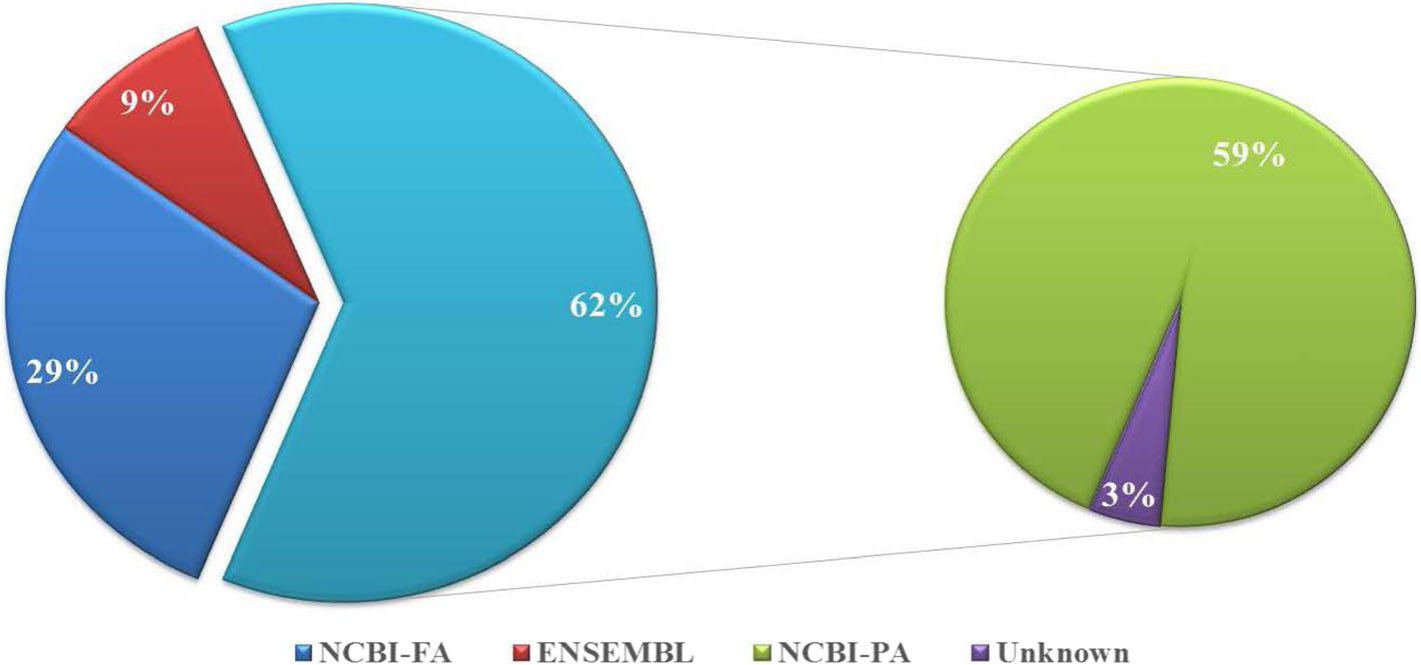
Summary of the boar sperm proteome. Total of 2728 proteins were identified using shotgun method, with 781 fully (NCBI-FA) and 1625 partially (NCBI-PA) annotated through NCBI-nr, 238 annotated with ENSEMBL and 84 that remained unknowns

Nearly 90% concordance was found amongst individual proteome dataset (*n* = 8). A total of 2728 unique and commonly shared proteins were identified with confidence among samples (*P* ≤ 0.05; see Additional file 2 Total proteins). Total of 2406 detected proteins were annotated with NCBI-nr databases, corresponding to 781 (29%) full and 1625 (59%) partial annotations. Residual proteins showed 238 (9%) annotations with ENSEMBL and 84 (3%) that remained unknown (Fig. 1). With an arbitrary cut-off set at *P* ≤ 10^− 99^, 116 proteins were delimited and considered highly abundant (Additional file 2 Abundant proteins). Of these abundant proteins, 61 were fully annotated (NCBI-nr) with many of them deriving from testes (i.e., fascin-3 and epididymal sperm-binding protein 1) and sex glands (i.e., seminal plasma protein pB1, plasma sperm motility inhibitor, and angiotensin I converting enzyme). Number of abundant proteins appeared associated with sperm structure (i.e., protamine-1 or PRM-1, several tubulin family members, F-actin capping protein β1 and 2, and outer dense fiber protein or ODF1) and sperm-egg interactions (i.e., sperm acrosome membrane-associated protein 1 or SPACA1, sperm equatorial segment protein 1 or SPESP1, sperm adhesion molecule 1 or SPAM1, and spermadhesins (AWN, PSP-I/II, and AQN-3 or SPMI).

### Total protein identification using 2-DE

A total of 2123 spots were shared among all boar samples (*n* = 8). Gel electrophoreses (Additional file 3: Figure S2) usually displaying likely bimodal protein distributions based upon their isoelectric points (from ~ 5.0 to 7.5 and from ~ 8.0 to 10) and their molecular weights (from ~ 27 to 80 kDa and from ~ 21-10 kDa). Eight randomly selected spots were identified through MS/MS as albumin (69 kDa - pH 5.8), PSP-I (12 kDa - pH 7.8), ODF1 (29 kDa - pH 8.4), acrosin inhibitor (11 kDa - pH 8.9), F-Actin capping protein subunit beta (31 kDa - pH 5.5), AWN (17 kDa - pH 9.3), lactadherin precursor (48 kDa – pH 6.3), and triosephosphate isomerase (27 kDa - pH 6.5).

### Shotgun protein validation through immunotechniques

**Fig. 2:**
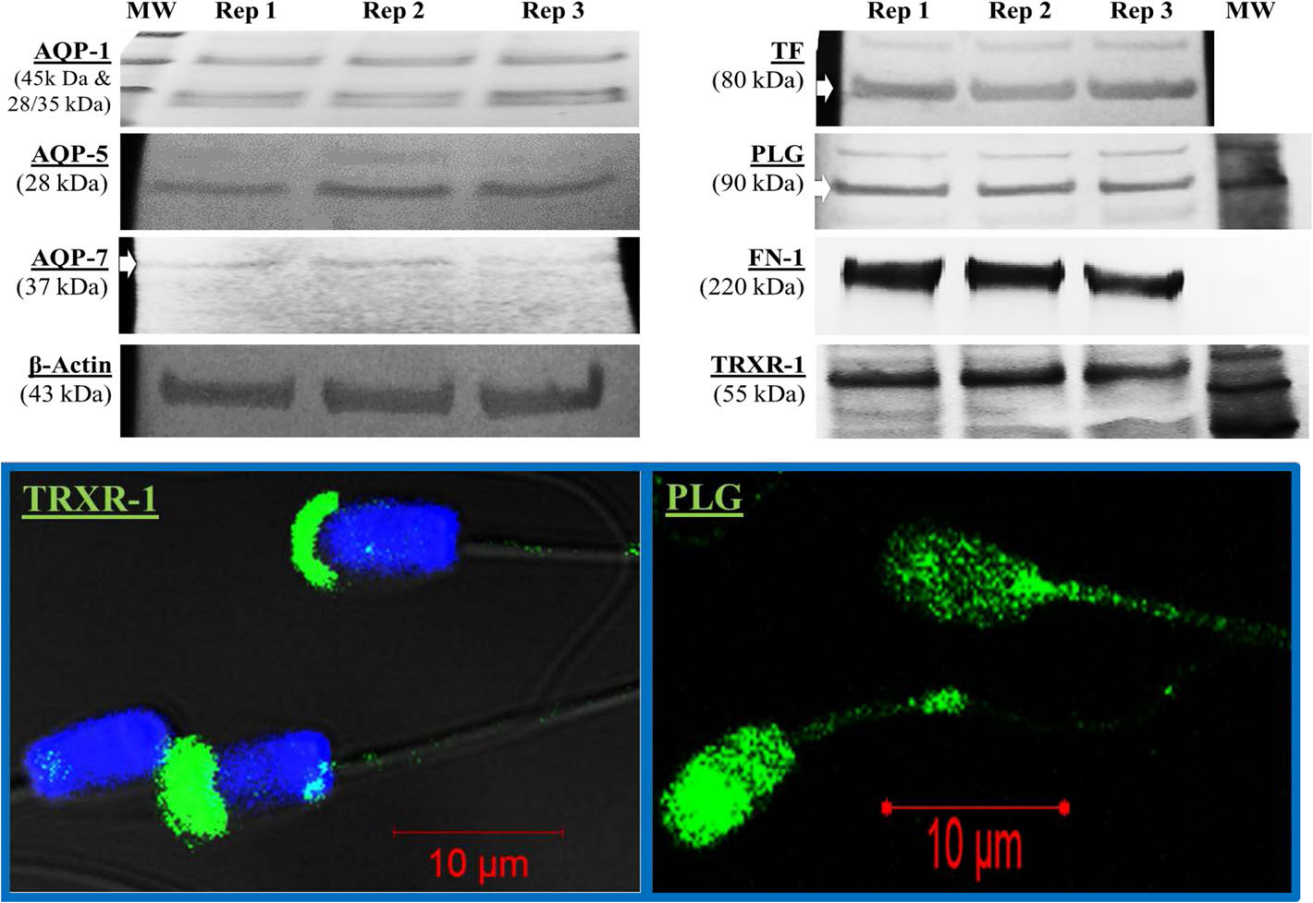
Representative westernblots and *in situ* immunofluorescence of boar spermatozoa. Selected proteins were targeted with antibodies raised against human AQP-1 (45 kDa & 28/35 kDa), AQP-5 (28 kDa), AQP-7 (37 kDa), β-actin (43 kDa, the internal loading control), TF (80 kDa), PLG (90 kDa), FN-1 (220 kDa), TRXR-1 (55 kDa). In situ immunofluorescence micrographs of TRXT-1 and PLG are also shown. The FITC green fluorescence indicates the sub-apical (acrosome membrane) localization of TRXR-1 on the sperm head and the localization of PLG on the head and mid-piece of spermatozoon. Nuclei are counterstained in blue with DAPI

**Fig. 3:**
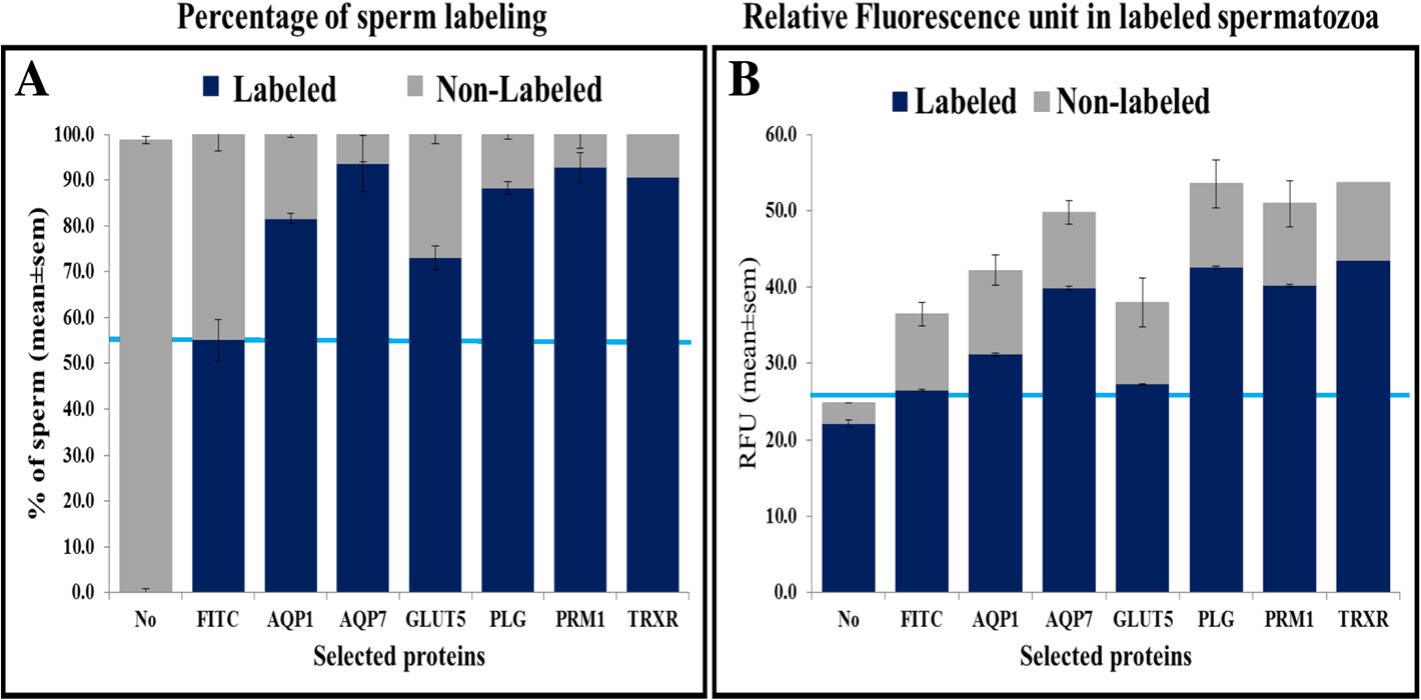
Immunofluorescence quantification through flow cytometry. Columns labeled as “No” or “FITC” correspond to spermatozoa incubated without any antibodies or with FITC-conjugated secondary antibody only, respectively. **a** Percentage of immunopositive sperm cells and **b** Relative fluorescence intensity of labeled spermatozoa. Specific targeting is revealed above the blue line, suggesting for example for GLUT-5 detection, a lower proportion of cells being targeted (**a**) and which cells also show lowest or no specific detection of GLUT-5 (**b**)

Aquaporins (AQP-1 and AQP-5), aquaglyceroporins (AQP-7), transferrin (TF), plasminogen (PLG), fibronectin-1 (FN-1), and thioredoxin reductase 1 (TRXR-1) were immunodetected in sperm protein lysates (Fig. 2). With comparable protein loads into electrophoresis wells, signal bands of AQPs appeared weaker than that of TF, FN-1 and TRXR-1. These proteins together with GLUT-5 and PRM-1 were targeted using **in situ** immunofluorescence, followed by confocal microscope imaging (Fig. 2); thus differential fluorescence intensities and localizations of proteins on spermatozoa were revealed. Representative indications of these differences are shown in Fig. 2, with TRXR-1 exhibiting a strong signal located in the apical (acrosome) region of the sperm head. In parallel, the PLG signal was mainly detected on the head and mid-piece regions of the spermatozoon. Additionally, flow cytometry revealed high proportions (≈70-90%) of spermatozoa immunoreacting with anti-AQP-1, -AQP-7, -GLUT-5, -PLG, -PRM-1, and -TRXR-1 antibodies (Fig. 3a) and fluorescence intensities significantly above the thresholds obtained with control spermatozoa incubated without antibodies (No) or with the FITC-conjugated secondary antibody only (Fig. 3b). The fluorescence intensities of sperm alone (non-stained or control; No) and sperm incubated with the secondary antibody only (FITC) can be seen in Fig. 3.

### Validation through gene expression

**Fig. 4:**
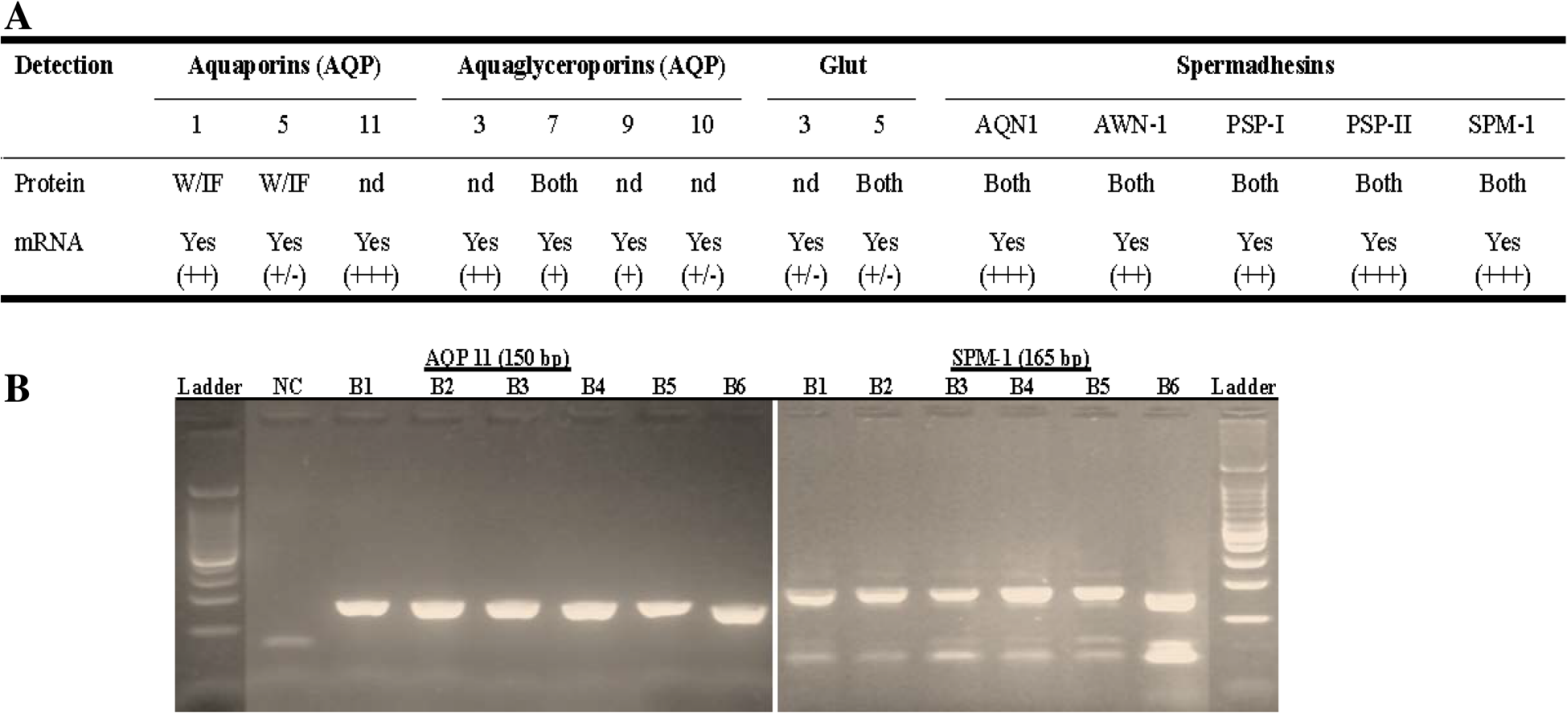
Protein and mRNA transcript comparison for selected proteins. **a** Table summarizes: protein detection with westernblotting (WB), immunofluorescence (IF), or altogether with shotgun approach (Both) and relative intensities of PCR amplicon bands classified as strong (+++), mid (++), weak (+), and low (+/−). **b** Representative gel electrophoreses of AQP11 and SPMI (AQN-3) of six boars (B1-B6) are shown. NC = Negative control and Ladder with band size starting from 100 bp (100 bp increment). On the SPMI gel, the arrow-head indicates the position of the expected amplicon size while the second band correspond to primer dimers

Aquaporin (AQP-1, AQP-5, and AQP-11), aquaglyceroporins (AQP-3, AQP-7, AQP-9, and AQP-10), glucose transporters (GLUT-3 and GLUT-5), and spermadhesins (AQN-1, AWN1, PSP-I, PSP-II, SPMI) were targeted in this study. Data summarized in Fig. 4a indicate the detection of mRNA transcripts (Yes) in spermatozoa. Although similar amounts of cDNA were used for PCRs, the amplicon bands of each gene appeared at variable intensities as seen on the gel electrophoresis allowing for a relative classification, from weak (+/−) to strong (+++) for each gene target. In the current study, all spermadhesin family members and AQP-11 signal bands were the strongest, and Fig. 4b shows a representative agarose gel electrophoresis of AQP-11 and SPMI amplification products. Fig. 4a also indicates the status of protein detection of each targeted gene in boar spermatozoa by either westernblotting (W) or immunofluorescence (IF).

### Functional analyses

#### Total proteins

**Fig. 5:**
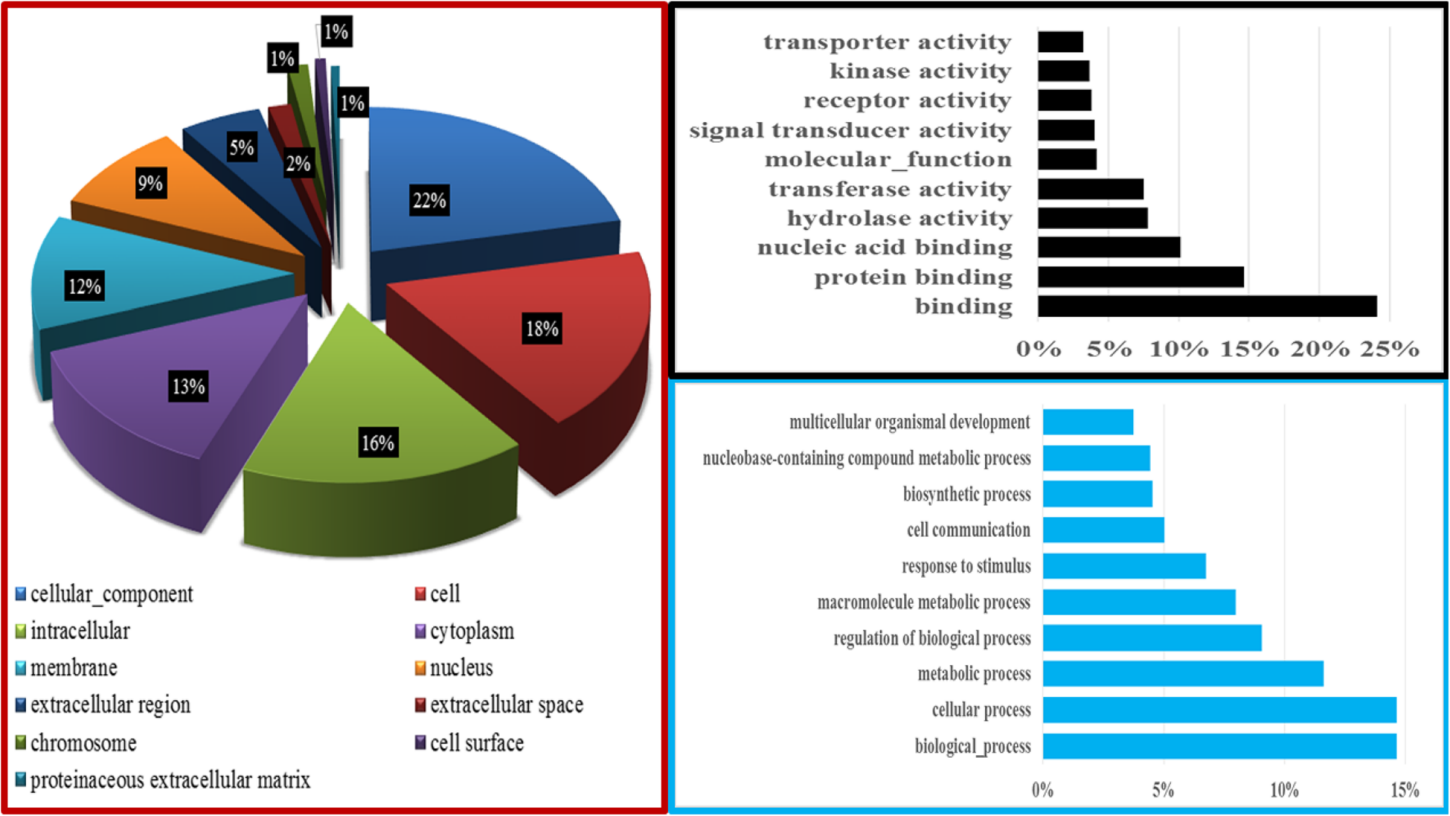
Functional annotation of the boar sperm proteome. The distribution of total protein per gene ontology (GO) terms in the Cellular Component is shown in the red frame. The top 10 GO terms in the Molecular Function and Biological Process categories are shown in the black and the blue frames, respectively

Analyses were performed on the shotgun dataset only and data with P (Benjamini-Hocheberg) ≤ 0.05 and FDR ≤ 10% were retained for interpretations. A systematic search for compatible pig protein identifiers resulted in 2236 successful conversions (using GO*Retriever* from Agbase), corresponding to 82% of the total sperm proteome being used for functional classification of all GO terms (Fig. 5). Detailed distributions of the biological functions are categorized per cellular compartmentalization (CC, with 2070 annotations), molecular function (MF, with 5659 annotations), and biological processes (BP, with 15,402 annotations).

For enrichment studies, the dataset with pig identifiers yielded only 39% conversions for DAVID analyses. However, the optimal conversion of 77% (2108 proteins) was reached by combining the residual unconverted pig identifiers with their human homologs, representing 67 *Sus scrofa* plus 2041 *Homo sapiens* proteins that were considered for analyses.

Totals of 67, 38, and 92 GO terms were found significantly enriched (*P* < 0.05) within the CC, MF, and BP categories, of which 34, 20, and 24 had a FDR ≤ 10%, respectively (Additional file 2 Total protein_FA). In comparison to GO term classifications by Agbase, terms associating mitochondria, extracellular matrix, and cytoskeleton in total proteins were significantly enriched was enriched (1.4× - 2.5×) in the CC category. Likewise, most GO terms (i.e., catabolic, transferase, and hydrolase activities and nucleic acid, protein binding and binding) highly classified in Agbase (Fig. 5) were significantly enriched through DAVID, in the MF category. Finally, GO terms associating response to chemical stimulus and biological, cellular, and metabolic processes in BP were significantly enriched (≈ 1.1× – 1.5×), and those associated with reproduction (i.e., sexual reproduction and reproduction) and oxygen consumption (i.e., oxidative phosphorylation, cellular respiration, and aerobic respiration) appeared amongst the highly and significantly enriched (> 1.4× – 3.6×) in the proteome dataset.

#### Abundant proteins

A total of 62% of proteins (71) were successfully converted to DAVID for analyses. Proteins associated with protein complex, cytoskeleton/cytoskeletal parts, and organelle GO terms were significantly expressed (3-9×) in CC category. Enrichments (> 12×, *P* < 0.05) were found in favor of fertilization and reproduction GO terms in the BP category and proteins such as seminal plasma protein pB1, milk fat globule EGF factor 8 protein, epididymal sperm binding protein1, and sperm associated AWN protein, PSP-I/II, sperm adhesion molecule 1, zonadhesin, and zona pellucida binding protein are among the fertilization GO term (Additional file 2 Abundant protein_FA).

### Pathway analyses

The KEGG pathway analysis of the total proteome revealed 21 hits, of which only oxidative phosphorylation, citrate cycle (TCA cycle), and ECM-receptor interaction pathways were significantly enriched, with respectively 35, 13, and 23 protein counts for each (Additional file 2 Total protein_FA). Enrichment folds varied from 2.0 to 3.2×, with Benjamini *P* values ranging from 0.004 to 0.032 and corresponding FDR from 0.05 to 1.09. Only the gap junction pathway was significantly overrepresented in the abundant protein list, with a fold enrichment of 11.8× and Benjamini P and FDR values of 0.0045 and 0.087, respectively. This pathway comprised six proteins that included tubulin α1a, tubulin β1, and tubulin α3 (Additional file 2 Abundant protein_FA).

## Discussion

The full characterization of the global sperm proteome has the potential to provide new clues to better understand the genesis and maturation of spermatozoa and to lead to concrete improvement of reproduction outcomes. Here we used two common high-throughput techniques that have overlapping strengths and weaknesses [24, 27] for a comprehensive proteome profiling of boar spermatozoa to provide a unique dataset for further in-depth investigation and improvement of post-collection boar semen handling.

The use of the shotgun technique in this study provides the largest pool of boar sperm proteome (2728 total proteins) that falls into the current range reported in spermatozoa (~ 1000 to ~ 4000) of various species such as cow, horse, human, mice, and rat [25, 26, 46]. The study confirms the power of the shotgun (LC-MS/MS) over the 2-DE (2D-MS/MS) method to generating greater number of proteins. Nonetheless, our 2-DE method yielded more protein spots (2123) than the unique reported number (1577) in boar spermatozoa [47].

Proteomic investigations are continuously and consistently showing discrepancies in the numbers of detected proteins between shotgun and gel-based proteomics [24, 27]. This situation can be caused by sample preparation procedures, cut-offs in data acquisition and processing, as well as other factors known to limit the capacity of the 2-DE gels. For example, the presence of abundant proteins on the 2-DE gel compete for space and mask or prevent the detection of neighboring protein spots, while low expressed or less represented protein spots, as seen here with proteins within the 27-80 kDa range (Additional file 3: Figure S2), may not be considered for analyses. Nonetheless, the gel-based proteomic (e.g., 2D-DIGE-LC/2MS) remains the main technique used for comparative studies performed with boar spermatozoa [33, 47–49], while the speed of discovery could be accelerated by the shotgun approach providing a panoramic proteome profile to allow for more complete comparative studies.

With 97% total protein being partially annotated, the current dataset provides a vast reservoir of proteins for further investigation and characterization of boar spermatozoa. Still, the shotgun proteomic is a bottom-up holistic approach that needs further validation of detected proteins. Both immunotechniques and gene expression led to some conflicting findings, particularly with the immunodetection of proteins that are not found in the generated proteome dataset (e.g., PRM-1, AQP-1, and AQP-5) or already reported elsewhere by other authors. For example, previous studies have detected the epididymis secretory glutathione peroxidase or GPX5 [50] and AQP-11 [51] in their small-scale studies using westernblotting in pig spermatozoa. These two proteins are not present in our dataset, where conflicting findings may be due to the stringency of peptide number cut-off for the calling of proteins. In the present study, reported proteins are from peaks of at least three peptides that were shared among the MS/MS runs of all individual samples (*n* = 8). It is therefore, likely that many proteins may have been filtered out in the current study, resulting in fewer observations as suggested in a previous report [27]. Furthermore, the gene expression study confirmed the presence of selected RNA transcripts in spermatozoa, including those of proteins not found in our study (e.g., AQP-1 and AQP-5). In sum, these findings highlight one of the disadvantages or limits of the shotgun proteomics, mainly for biomarker discovery [51, 52].

On the other hand, many known proteins such as spermadhesins and triosephosphate isomerase that are already reported in the literature were found in the current dataset [47, 53]. Therefore, with 29% (791) of the total protein being fully annotated, the current dataset constitutes an important platform for future annotation updates that may lead to even greater numbers of proteins that will likely provide new research opportunities to enhance our comprehension of the biology of boar spermatozoa.

Meanwhile, the investigation of highly abundant proteins has the potential to boost the knowledge needed to make further progress in assisted reproduction. Numbers of these proteins are known to play crucial roles in 1) protecting spermatozoa from the acidic pH and antimicrobial immune responses of the vagina, 2) assisting spermatozoa during the cervical mucus transit [54, 55] or 3) maintaining the function of spermatozoa until interactions with the oocyte [11]. Especially, spermadhesins have been reported as the major proteins of boar seminal plasma. Their expression has been detected in vesicular glands, epididymis and rete testis of boars while their secretions are found in the male genital tract and seminal plasma [11, 12, 52, 56, 57]. The presence of spermadhesins on sperm membrane and possible effects on sperm function, from motility to interaction with the oocyte, have been reported [11, 58, 59]. In addition to the current knowledge, our study brings further support to the small number of studies identifying the presence of heparin (AQN-1, AQN-3, and AWN-1) and non-heparin (PSP-I and PSP-II) spermadhesin mRNA transcripts in mature boar spermatozoa [52]. In the current dataset, AQN-3 was identified as sperm motility inhibitor or SPMI, which is also known to bind plasma membrane of boar spermatozoa and act as sperm receptor of the oocyte zona pellucida protein [60]. More interestingly, three major proteins associated with acrosome vesicle (acrosin, zona pellucida binding protein, and acrosin binding protein) and having important roles during sperm-egg interactions [61–63] are among the abundant proteins. The roles of many abundant proteins on sperm function are yet to be determined, and the functional analysis is giving direction into which individual or group of proteins may deserve in-depth investigation of their roles.

We found that numerous proteins were overrepresented (> 1.1× – 3.6×) in GO terms such as response to chemical stimulus and biological, cellular, and metabolic processes, oxidative phosphorylation, cellular respiration, an aerobic respiration, thus supporting a concerted effort of various pathways to regulate sperm function, especially motility. In this study, oxidative phosphorylation (vs. glycolysis) is coming up as the key or highly enriched metabolic pathway supplying the high demands for ATP, necessary to achieve progressive motility and more. However, experimental studies have shown the glycolytic pathway as the primary source of energy for sperm motility in many species, including pigs [64–67]. The likely contribution of oxidative phosphorylation to sperm motility may not be ruled out, as the detection of enzymes such as adenylate kinase and phosphoglycerate kinase that shuttle ATP away from the mitochondrion to the flagellum are suggesting the possible role of oxidative phosphorylation.

We also found that the gap junction pathway was highly and significantly represented within the in abundant proteins dataset. Gap junctions are intercellular channels relying on a family of protein membranes known as connexins. These molecules couple the cytoplasms of interacting cells to allow passage of ions and other small molecules [68]. Here, the gap junction enrichment together with the aforementioned “response to chemical stimulus” should not be a surprise given the variety of sperm interactions with their surroundings (i.e., fluids and uterine and oviduct cells). Indeed, interactions with chemical stimuli such as K^+^, Na^+^, Ca^2+^, and pH can leads to physiological events such as metabolic transport and signaling, capable to induce contractions in sperm assemblies resulting in motility. Studies have reported the capability of connexins 36 and 43 to anchor microtubules consisting of alpha and beta tubulin proteins dimerization [69]. Therefore, the abundance of tubulin proteins and their classification as part of the gap junction could be seen as a good indicator of their role in sperm motility.

Additionally, significant and high enrichments (> 12×) in favor of fertilization and reproduction GO terms were observed among abundant proteins; however, the full roles of associated proteins in sperm function remain to be determined [1].

### Practical implications

Overall, the present study gives a panoramic list of proteins for further identification of biomarkers associated with various physiological traits of the male, which will be of interest for agricultural and translational purposes [1, 12]. The current dataset confirms the presence of proteins (e.g., acrosin-binding protein, fibronectin 1, and triosephosphate isomerase) that have been proposed as potential predictors of semen freezability in previous studies [12, 32, 47, 49, 70]. Various glucose transporters, and aquaporin family members playing crucial roles during cell membrane permeability to water (aquaporins) and cryoprotectants (aquaglyceroporins) were detected. These findings contribute to the growing investigation of their roles in sperm function [51, 53, 71, 72], while the apparent weaker detection levels of aquaglyceroporins may be consequential to boar sperm freezability [73].

## Conclusions

The proposed shotgun proteomics method is the first employed in boar spermatozoa and has allowed the identification of over 2 thousand proteins for a comprehensive bioinformatic analysis of their biological functions. Findings confirmed previous reports using different investigative methods and provide a large reservoir for further search of valuable biomarkers to improve assisted reproductive technology outcomes. Yet, the generated dataset is subjected to continuous update with future annotations to determine the functional significance of detected proteins for more insights into the male (in) fertility.

## Additional files

Additional file 1: Figure S1. Sperm preparation and proteomics. (PDF 153 kb)

Additional file 2: Shotgun proteome data set analyses. File contains the total and abundant proteins, with associated functional analyses (Total protein_FA and abundant protein_FA). (XLSX 417 kb)

Additional file 3: Figure S2. Representative two-dimensional gel electrophoresis of the boar sperm proteome. (PDF 84 kb)

## Abbreviations

2D-MS/MS: Two-Dimensional coupled with tandem Mass Spectrometry (MS/MS)
ARTs: Assisted reproductive technologies
BP: Biological process
CC: Cellular component
ECM: Extracellular matrix
GO: Gene ontology
KEGG: Kyoto enclyclopedia of genes and genomes
MF: Molecular function
mRNA: Messenger ribonucleic acid
NCBI–nr: National Center for Biotechnology Information non-redundant
PBS: Phosphate-buffered solution
SDS-PAGE: Sodium Dodecyl Sulfate -PolyAcrylamide Gel Electrophoresis
TCA: The Citric Acid or TriCarboxylic Acid cycle
