# Supplementary material for: In-depth proteomic analysis of boar spermatozoa through shotgun and gel-based methods
Source: BMC Genomics. 2018 Jan 18;19:62. doi: 10.1186/s12864-018-4442-2 (PMC5774113; doi:10.1186/s12864-018-4442-2)
Supplement: Supplementary file 1 — Sperm preparation and proteomics. (PDF 153 kb) [file 12864_2018_4442_MOESM1_ESM.pdf]

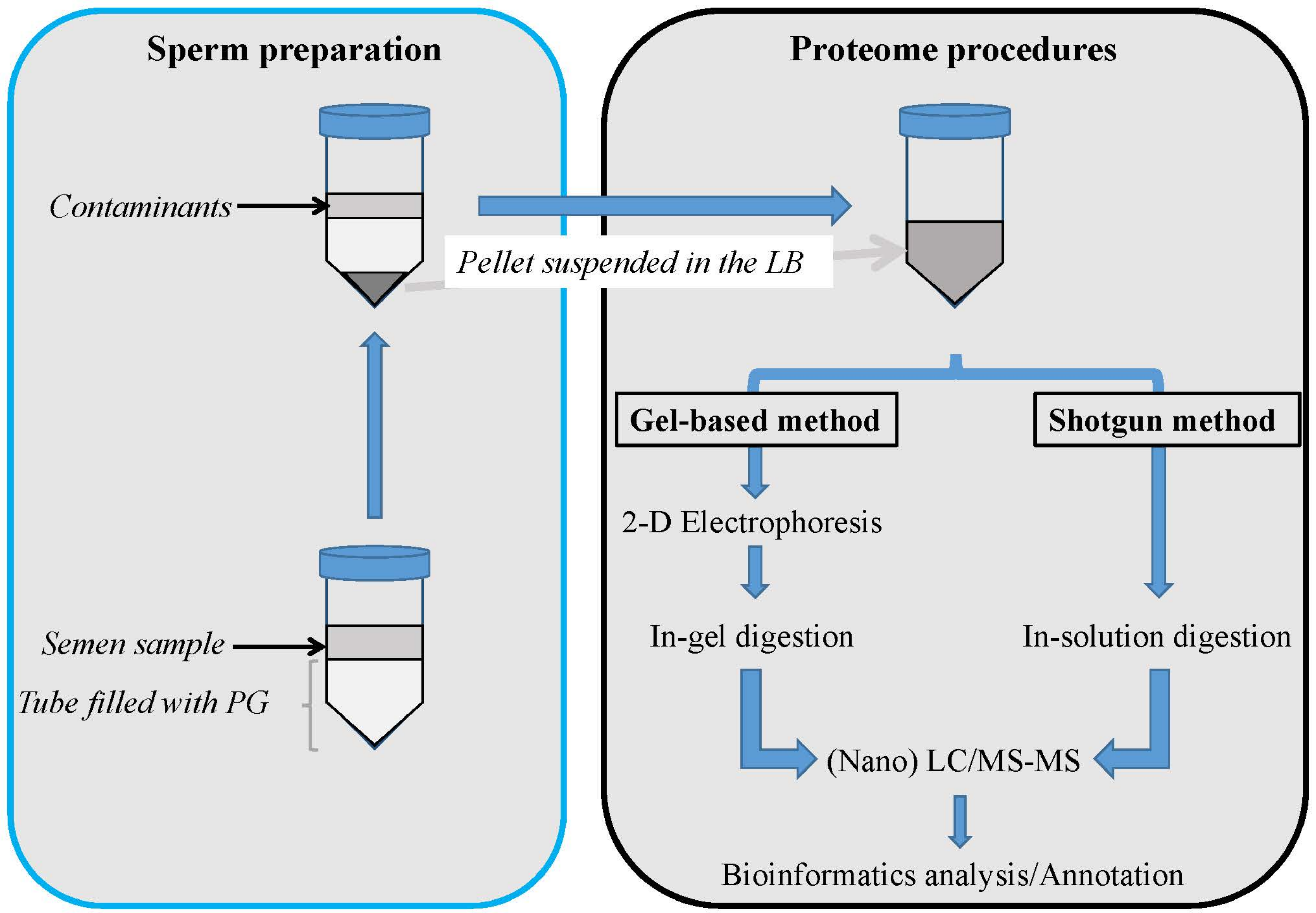
Sperm preparation
Contaminants
Pellet suspended in the LB
Semen sample
Tube filled with PG
Proteome procedures
Gel-based method
Shotgun method
2-D Electrophoresis
In-gel digestion
In-solution digestion
(Nano) LC/MS-MS
Bioinformatics analysis/Annotation
