# Supplementary material for: In-depth proteomic analysis of boar spermatozoa through shotgun and gel-based methods
Source: BMC Genomics. 2018 Jan 18;19:62. doi: 10.1186/s12864-018-4442-2 (PMC5774113; doi:10.1186/s12864-018-4442-2)

1. Albumin
2. Lactadherin precursor
3. F-actin capping protein subunit beta
4. ODF-1
5. Triosephosphate isomerase
6. AWN
7. PSP-I
8. Acrosin Inibitor

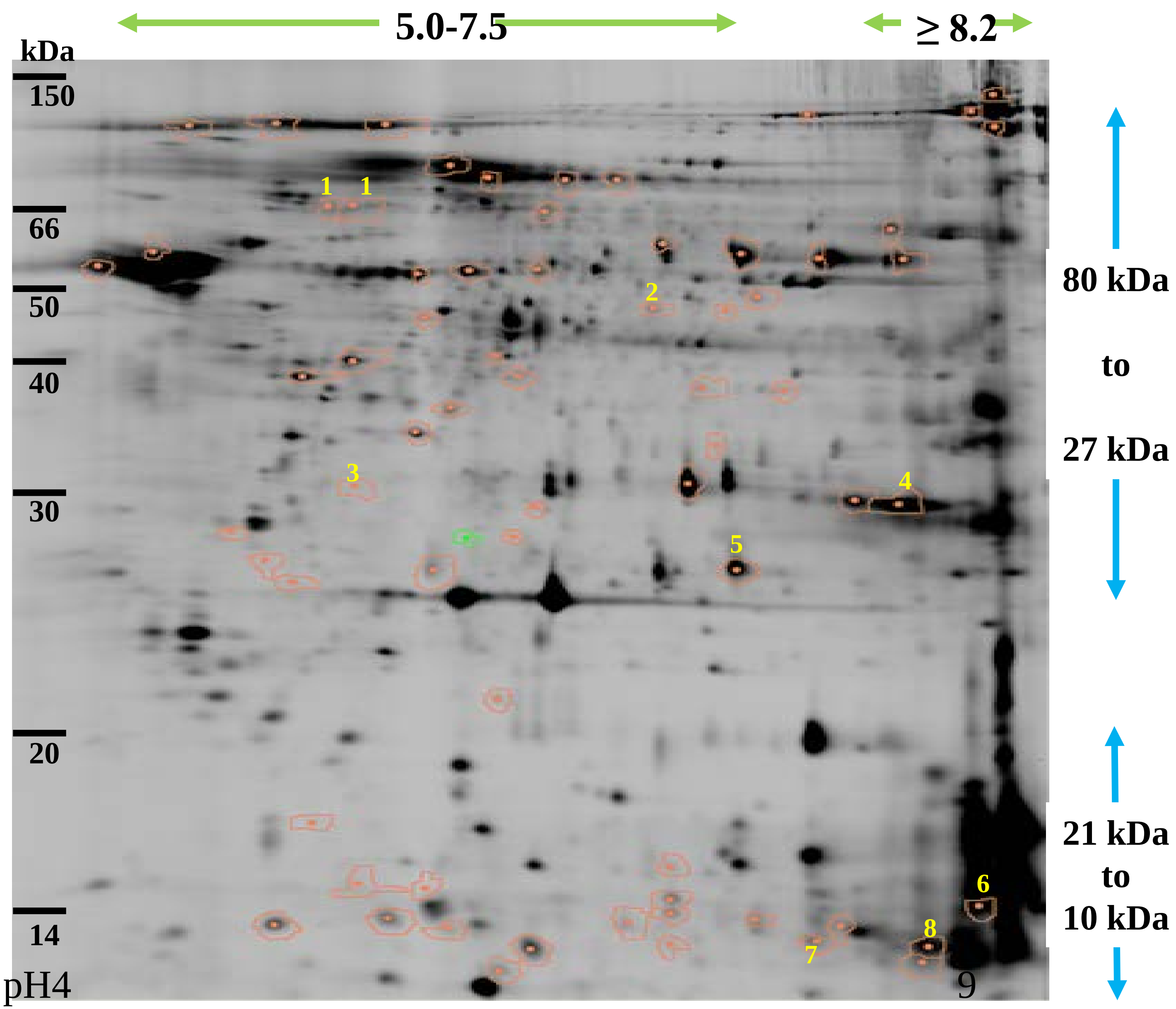

Supplement: Supplementary file 3 — Representative two-dimensional gel electrophoresis of the boar sperm proteome. (PDF 84 kb) [file 12864_2018_4442_MOESM3_ESM.pdf]
